# Dynamic trajectories of sepsis-induced immunoparalysis: from biological mechanisms to precision monitoring and targeted intervention

**DOI:** 10.3389/fimmu.2026.1933094

**Published:** 2026-10-16

**Authors:** Chengxin Xue, Yiheng Hu, Xinyun Wu, Yudan Chen, Zihao Zhou, Qidong Jiang, Bo Yang, Chengli Wen

**Affiliations:** 1 Department of Critical Care Medicine, The Affiliated Hospital, Southwest Medical University, Luzhou, China; 2 School of Clinical Medicine, Southwest Medical University, Luzhou, China; 3 School of Medical Imaging, Southwest Medical University, Luzhou, China; 4 School of Public Health, Southwest Medical University, Luzhou, China; 5 Department of Gastroenterology, Xuyong County People’s Hospital, Luzhou, Sichuan, China

**Keywords:** biomarkers, dynamic trajectories, immune monitoring, immunoparalysis, precision immunotherapy, sepsis

## Abstract

Sepsis is increasingly recognized as a heterogeneous syndrome in which profound immunosuppression, or immunoparalysis, may coexist with or follow the initial hyperinflammatory response. This state is characterized by impaired host responsiveness to pathogens and secondary immune stimuli and is associated with secondary infections, persistent organ dysfunction, and increased mortality. This narrative review summarizes the conceptual evolution and biological basis of sepsis-induced immunoparalysis, with particular emphasis on innate immune deactivation, adaptive immune dysfunction, immune-checkpoint upregulation, and metabolic-epigenetic reprogramming. A central focus is the transition from static, single-time-point assessment toward longitudinal characterization of immune trajectories. Current evidence suggests that the first 1–2 weeks after sepsis onset represent a critical period during which heterogeneous recovery patterns become apparent. Empirically supported patterns include improving or recovering immune states and persistent hyporesponsiveness, whereas fluctuating transitions remain a proposed, hypothesis-generating trajectory requiring further longitudinal validation. We review major monitoring indicators, including monocyte human leukocyte antigen-DR (mHLA-DR), absolute lymphocyte count (ALC), ex vivo cytokine production capacity, and immune checkpoints such as programmed death-1 (PD-1) and programmed death-ligand 1 (PD-L1). Importantly, most currently available biomarkers have stronger evidence for immune phenotyping and prognostic assessment than for predicting treatment response, and reported thresholds remain dependent on assay methodology, patient population, and measurement timing. We further discuss emerging biomarker-guided approaches, including the ImmunoSep trial, as early proof-of-concept for phenotype-enriched immunotherapy rather than definitive evidence for routine clinical implementation. Overall, repeated multi-marker assessment may improve characterization of immune trajectories and help identify candidate treatment-eligible windows; however, biomarker-based treatment thresholds, composite immune-clinical frameworks, and phenotype-matched immunotherapies require further prospective validation, particularly with respect to mortality, safety, and other patient-important outcomes.

## 1 Introduction

### 1.1 Pathophysiological background of sepsis-induced immunoparalysis

Sepsis is a life-threatening organ dysfunction caused by a dysregulated host response to infection (1). Traditionally, sepsis has been viewed as a syndrome driven predominantly by excessive inflammation, often described as a “cytokine storm.” However, this paradigm has been increasingly challenged. Accumulating evidence indicates that sepsis is not solely a hyperinflammatory condition but rather a highly heterogeneous syndrome in which profound immunosuppression may coexist with or even follow the initial inflammatory response (2–4).

In a substantial proportion of patients, impaired immune responsiveness can occur early in the disease course, a condition commonly referred to as immunoparalysis, characterized by a markedly reduced ability of the host to respond to pathogens and secondary immune stimuli (2, 5). This immunosuppressive state has been closely associated with an increased risk of secondary infections, persistent organ dysfunction, prolonged intensive care unit (ICU) stay, and higher mortality, and is now recognized as a critical determinant of sepsis outcomes (5–9).

With the advancement of longitudinal studies, increasing evidence suggests that immune dysfunction in sepsis should not be considered a static phenomenon but rather a dynamic process evolving over time. Conventional studies have relied on single time-point measurements of immune markers; however, such approaches fail to capture temporal heterogeneity (7, 10). Recent prospective studies have demonstrated that key immune parameters—including mHLA-DR expression, absolute lymphocyte counts, and ex vivo cytokine production capacity—undergo significant time-dependent changes during the first 1–2 weeks following sepsis onset, with heterogeneous recovery trajectories across patients (11–16). For example, mHLA-DR expression may exhibit persistent suppression, gradual recovery, or fluctuating patterns, and its recovery kinetics are closely associated with clinical outcomes (11, 12, 14). Similarly, lymphocyte counts and cytokine production capacity also demonstrate variable dynamic changes (7, 12, 15). Moreover, differences in immune trajectories have been observed among patients with distinct infectious etiologies, further highlighting the heterogeneity of the host response in sepsis (13, 16). Collectively, these findings suggest that immune status should be regarded as a dynamic process evolving over time, rather than a static measurement at a single time point.

Based on this evolving understanding, the present review aims to provide a comprehensive overview of the dynamic trajectories of sepsis-induced immunoparalysis and its monitoring strategies. Specifically, we summarize the conceptual framework and biological basis of immunoparalysis, review current evidence on dynamic immune trajectories, discuss available immune monitoring biomarkers and integrated assessment approaches, and explore how immune monitoring may facilitate the development of precision immunotherapy in sepsis.

### 1.2 Methodology of the narrative review

This article was designed as a narrative review rather than a systematic or scoping review. Relevant literature was identified through searches of PubMed, Embase, Cochrane Library, Web of Science Core Collection from the establishment of each database to July 31, 2026, using combinations of terms including “sepsis,” “immunoparalysis,” “immunosuppression,” “mHLA-DR,” “lymphopenia,” “immune checkpoint,” “immune trajectory,” “immune monitoring,” “immunotherapy,” “endotoxin tolerance,” “sepsis-induced immunoparalysis,” and “macrophages.” Priority was given to human prospective and longitudinal cohort studies, randomized clinical trials, systematic reviews, and major mechanistic studies directly relevant to sepsis-induced immunoparalysis. Preclinical animal and *in-vitro* studies were included when they provided mechanistic information not yet available from human studies. Reference lists of key reviews and eligible articles were also screened for additional relevant studies. Because this was a narrative review, no formal meta-analysis or standardized risk-of-bias assessment was performed; however, the interpretation of evidence considered study design, population, sample size, directness of evidence, consistency across studies, and clinical relevance.

## 2 Concept and connotation of sepsis-induced immunoparalysis

### 2.1 Definition

Sepsis-induced immunoparalysis generally refers to a state of impaired immune responsiveness that occurs during infection, characterized by a markedly reduced ability of the host to respond to pathogens and secondary immune stimuli (2, 5). Several related terms have been used in the literature, including immunoparalysis, sepsis-induced immunosuppression, and immune low-response state, which partially overlap but emphasize different aspects of immune dysfunction (2–4).

Specifically, sepsis-induced immunosuppression broadly describes the overall downregulation of immune function, whereas immune low-response state emphasizes insufficient immune activation. In contrast, immunoparalysis highlights a functional impairment in host defense, particularly the diminished responsiveness to both primary and secondary challenges (2, 4, 5). Therefore, immunoparalysis reflects not only immune suppression but also a loss of immune competence.

Clinically, immunoparalysis is commonly assessed using multiple immunological markers. Among these, decreased mHLA-DR expression is a hallmark of impaired antigen presentation. Early studies demonstrated monocyte deactivation in septic patients, characterized by reduced HLA-DR expression that can be partially restored by immunostimulatory interventions (11). Subsequent studies further showed that persistently low mHLA-DR levels are strongly associated with poor outcomes (12, 14). In addition, persistent lymphopenia and impaired cytokine production capacity are also recognized as key features of immunoparalysis (6, 7, 15).

### 2.2 Immunoparalysis is not simply a late-stage phenomenon

The traditional model suggests a sequential transition from early hyper-inflammation to late immunosuppression. However, this concept has been increasingly revised. Current evidence indicates that pro-inflammatory and anti-inflammatory responses can coexist early in sepsis and dynamically interact throughout the disease course (2–4).

Furthermore, significant inter-individual variability exists in both the timing and magnitude of immune suppression, reflecting the intrinsic heterogeneity of sepsis (7, 16). Longitudinal studies have demonstrated that immune parameters do not evolve synchronously; instead, they follow distinct trajectories over time. For instance, mHLA-DR expression, lymphocyte counts, and cytokine production capacity may recover at different rates, and these trajectories are closely linked to prognosis (11–16). These findings suggest that immunoparalysis is not restricted to the late stage of sepsis, but rather represents a dynamic process that can occur early and persist throughout the disease course.

From a mechanistic perspective, immunoparalysis involves complex immune reprogramming, including impaired antigen presentation, lymphocyte apoptosis and exhaustion, and dysregulated immune signaling pathways (3, 6, 7, 17). Importantly, emerging evidence suggests that this immunosuppressive state is potentially reversible. Clinical trials have shown that immunostimulatory therapies such as granulocyte-macrophage colony-stimulating factor (GM-CSF) or interferon-γ (IFN-γ) can partially restore immune function in selected patients (18).

### 2.3 Clinical significance of immunoparalysis

Sepsis-induced immunoparalysis is closely associated with adverse clinical outcomes. First, impaired immune responsiveness significantly increases the risk of secondary infections, as the host’s ability to eliminate pathogens and respond to new infections is compromised (6–8). Second, immunoparalysis is associated with persistent organ dysfunction. Dysregulated immune responses not only impair infection control but may also exacerbate tissue injury, thereby delaying recovery of organ function (3, 5). Clinically, immunoparalysis is associated with prolonged ICU stay and increased healthcare burden (8, 19). Most importantly, sustained immunosuppression—such as persistent low mHLA-DR expression or prolonged lymphopenia is strongly associated with increased mortality (7, 12, 14, 20). Therefore, immunoparalysis should be regarded not only as a marker of disease severity but also as a key pathophysiological mechanism influencing clinical outcomes.

## 3 Biological basis of sepsis-induced immune paralysis

Sepsis-induced immune paralysis is not driven by a single mechanism. It arises from functional imbalances across several levels, including innate immunity, adaptive immunity, metabolic regulation, and the negative immune regulatory network (2–5). The relative weight of each level varies between patients and forms the biological basis for the clinical heterogeneity of immune paralysis (3, 4). These levels also differ in their time scale. Some changes appear early in sepsis, whereas others accumulate slowly and determine whether immunosuppression persists. This difference in time scale is the mechanistic origin of the divergent recovery trajectories described in the next section, and the multi-layered biological mechanisms underlying sepsis-induced immunoparalysis are visually summarized in **Figure 1**.

**Figure 1:**
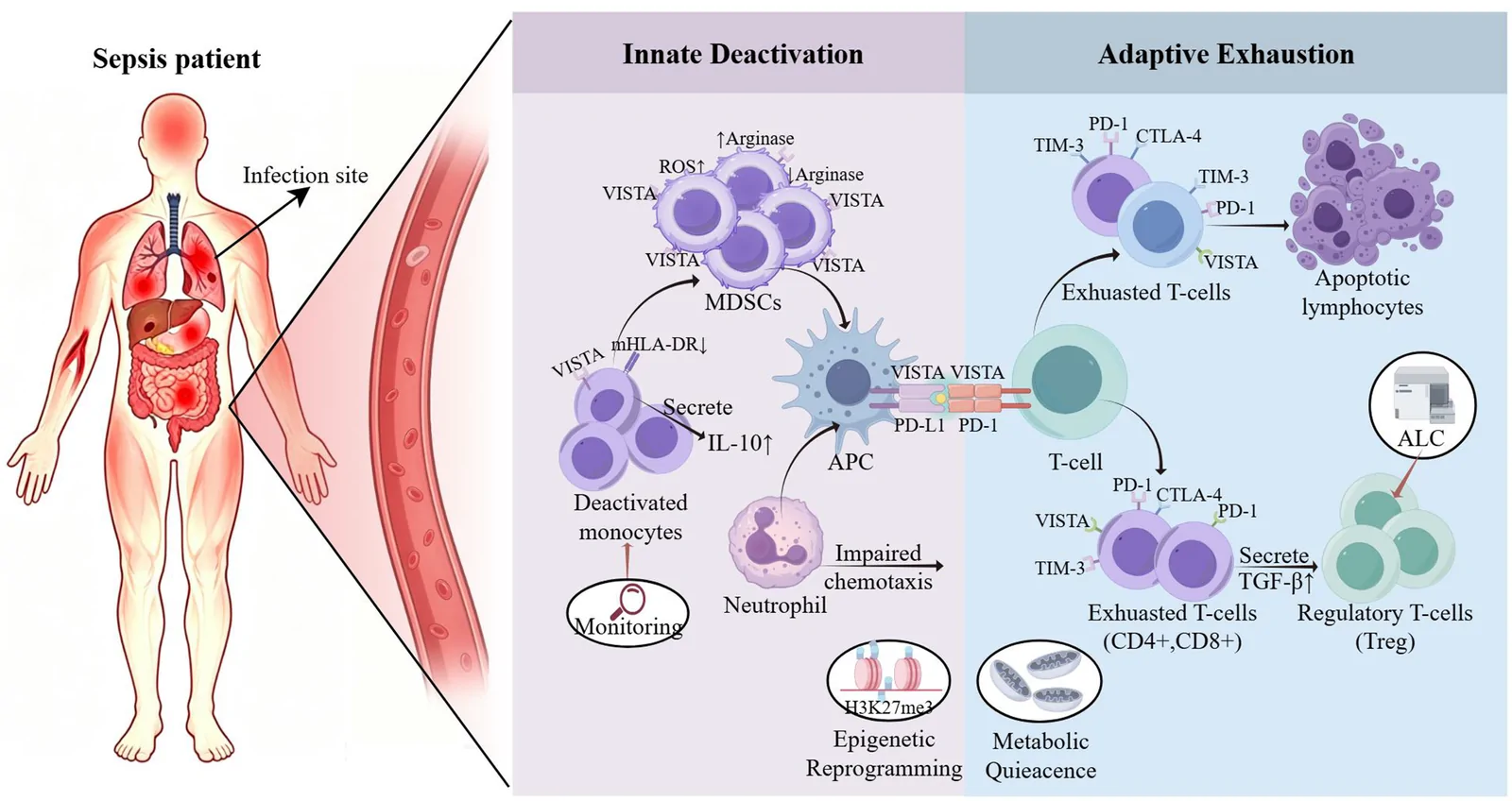
The dynamic evolution and biological mechanisms of sepsis-induced immunoparalysis. (By Figdraw).

### 3.1 Innate immune hyporesponsiveness

Monocyte-macrophages are the innate subpopulation most affected in sepsis-induced immune paralysis (2, 5, 21). During sepsis, circulating monocytes may lose antigen-presenting function and produce fewer proinflammatory cytokines (11, 21). This deactivated state is not random functional depletion but an actively regulated phenotypic shift. In deactivated monocytes, transcription of antigen presentation genes, including HLA-DR and the costimulatory molecules CD80 (B7-1) and CD86 (B7-2), is broadly suppressed, while anti-inflammatory molecules such as IL-10 and CD163 are maintained or upregulated (2, 5, 11, 21). The result is an asymmetric functional remodeling rather than uniform loss of activity.

Downregulation of monocytic HLA-DR (mHLA-DR) is the most widely used biomarker of innate immune status (5, 22, 23). Under Quantibrite-based assays, reported reference ranges in healthy populations have been approximately 13,500 to 40,000 antibodies per cell (AB/C), depending on the assay and study population (24–26). In observational cohorts of critically ill adults with sepsis or septic shock, an mHLA-DR below 15,000 AB/C has been associated with greater disease severity, whereas values below 8,000 AB/C have been used in intensive care unit immunophenotyping protocols to identify severe immune dysfunction (24, 25, 27). However, these thresholds depend on specific assays and platforms and may vary across study populations, cohorts, and flow cytometry protocols. They should therefore not be interpreted as universally interchangeable definitions of immunoparalysis (23, 26, 27). Accordingly, mHLA-DR is best regarded as a marker of immune phenotype and prognosis rather than, by itself, a validated predictive biomarker for response to a specific immunostimulatory therapy (11, 12, 14, 18, 23, 27). Thresholds used as eligibility criteria in immunostimulatory trials have varied: an mHLA-DR below 8,000 AB/C was used in the GM-CSF trial, whereas the ImmunoSep trial defined immunoparalysis using fewer than 5,000 HLA-DR molecules on CD45/CD14 monocytes (18, 28, 29). Their use as trial inclusion criteria does not establish their predictive value for treatment response. Measurement timing is also critical for clinical interpretation. A single baseline assessment provides limited information compared with longitudinal trajectories, and persistent failure to recover mHLA-DR expression by days 3 to 7 may indicate sustained immune paralysis more robustly than an isolated early measurement (12, 14, 25, 30). In a 20-year study of 1,023 patients with septic shock, Monneret et al. reported a strong prognostic association between persistently low mHLA-DR and adverse outcomes (25).

Beyond antigen presentation, deactivated monocytes show a reduced ability to produce TNF-α, IL-6, and IL-12 after lipopolysaccharide (LPS) stimulation, while IL-10 secretion is relatively preserved (11, 31, 32). This produces a global imbalance between pro-inflammatory and anti-inflammatory output. Poehlmann et al. (33) further showed that myeloid dendritic cells and plasmacytoid dendritic cells also undergo long-term functional deactivation that remains incompletely recovered even by day 28. At the same time, the phagocytic activity, oxidative burst, and migratory capacity of monocytes are impaired to varying degrees, which weakens clearance of residual pathogens and secondary infections (2, 5). This hyporesponsiveness does not stem from a fall in monocyte numbers but from a phenotypic remodeling associated with persistent transcriptional and epigenetic reprogramming (4, 31, 32).

A second alteration parallel to monocyte deactivation is the delayed expansion of myeloid-derived suppressor cells (MDSCs). Monocytic MDSCs (M-MDSCs) are defined as CD14-positive cells with low HLA-DR expression, a phenotype that overlaps closely with low-mHLA-DR monocytes in sepsis (2, 5, 34). In many settings, deactivated monocytes and expanded suppressive myeloid cells represent closely overlapping populations. In a retrospective study of 301 patients with septic shock, Waeckel et al. (34) found that M-MDSCs were elevated during the first week after septic shock onset, while sustained high M-MDSC levels at days 6 to 8 were independently associated with ICU-acquired infections and 28-day mortality. This delayed persistence matches the slow rhythm of monocyte functional recovery, and points to a maintenance mechanism within the innate compartment that is independent of the initial inflammatory peak and aligned in time with the sustained phase of immunosuppression.

### 3.2 Adaptive immune suppression

Unlike the acute deactivation of innate immunity, adaptive immune suppression develops as a progressive and multicellular impairment (2, 6). Lymphopenia is the earliest and most consistent immunological change in sepsis (6, 15, 35). Within 24 hours of ICU admission, peripheral lymphocyte counts fall sharply and remain low for days to weeks (15, 35, 36). This is driven mainly by apoptosis rather than cellular redistribution (35, 36). Post-mortem studies by Hotchkiss et al. and Boomer et al. revealed marked apoptosis and depletion of CD4-positive T cells, B cells, dendritic cells, and other immune populations in lymphoid and peripheral tissues of patients who died of sepsis-induced immune suppression (6, 36). Apoptosis proceeds through combined activation of the mitochondrial (caspase-9) pathway and the Fas/FasL pathway (2, 36). Non-classical death pathways such as pyroptosis and ferroptosis have also drawn increasing attention in septic lymphocyte depletion in recent years (37, 38). Multiple prospective cohorts confirm that persistent lymphopenia is independently associated with the risk of secondary infection and long-term mortality (15, 23, 39).

Adaptive immune suppression also stems from functional exhaustion rather than cell loss alone (6, 40, 41). A multi-organ autopsy study by Boomer et al. provided direct human evidence [6]. In patients dying of sepsis-induced immunosuppression, PD-1 and PD-L1 expression was markedly elevated in the spleen and lungs, together with widespread impairment of T cell function [6]. Exhausted T cells show high expression of several inhibitory receptors, including PD-1, TIM-3, and LAG-3, with reduced proliferation, lower cytokine secretion, and diminished cytotoxicity (40–42). CD4-positive T cells, CD8-positive T cells, and regulatory T cells (Tregs) follow different exhaustion rhythms. CD8-positive T cell exhaustion is usually more profound, while the relative expansion of Tregs further suppresses effector T cells through secretion of TGF-β and IL-10 (2, 5, 6).

Work on B cells and natural killer (NK) cells in sepsis-induced immune suppression remains limited. Dong et al. (43) observed a reduction in B cell numbers and an imbalance in subpopulation distribution in patients with septic shock, together with reduced immunoglobulin production and depletion of resting memory B cells. Reduced NK cell cytotoxicity weakens control of latent virus reactivation, such as cytomegalovirus (CMV) and Epstein-Barr virus (EBV, 2, 20).

Viewed at the system level, innate deactivation and adaptive suppression are functionally linked (2, 5). When monocyte antigen-presenting capacity fails, T cell activation and clonal expansion lose their upstream drive, which accelerates the collapse of adaptive immunity. Lymphocyte apoptosis peaks within the first 24 hours of sepsis and is therefore an early, rapid event, while whether lymphocytes recover depends on downstream metabolic and epigenetic control (2, 6, 36). The suppressive state is triggered by acute inflammatory signals, but its persistence comes from regulation at the metabolic and epigenetic levels.

### 3.3 Active maintenance of immune paralysis by metabolic and epigenetic reprogramming

A deep coupling exists between immunometabolism and immune cell function (3, 4). Metabolic reprogramming accompanies immune activation and, through downstream epigenetic effects, also feeds back to shape the functional state of immune cells. Under physiological conditions, classically activated (M1) macrophage polarization is accompanied by a metabolic shift toward aerobic glycolysis (the Warburg effect). Rapid ATP generation and pentose phosphate pathway products support cytokine synthesis and phagosome formation, while succinate accumulation amplifies transcription of proinflammatory molecules such as IL-1β by stabilizing HIF-1α (3, 4).

Deactivated macrophages in late sepsis show a starkly different metabolic profile. Glycolysis declines, oxidative phosphorylation is impaired, and mitochondrial dysfunction becomes evident, leaving the cell in a state of metabolic sluggishness (3, 4, 31). This dormancy restricts the energy supply and thereby limits phagocytosis, antigen processing, and cytokine synthesis. Metabolic dysfunction involving both glycolytic and oxidative pathways has been described in sepsis-associated immune dysfunction (3, 4, 31, 32). Metabolic abnormalities extend beyond glucose. Lipid metabolism is similarly dysregulated. Intracellular lipid droplet accumulation and remodeling of lipid synthesis programs in deactivated myeloid cells are not passive changes in energy reserves but metabolic signals that help maintain the tolerogenic phenotype. Zhang et al. (44) showed in a sepsis model that SREBF1-mediated lipid reprogramming drives dendritic cell immune paralysis by coupling with endoplasmic reticulum (ER) stress, and that targeting the SREBF1-lipid-ER stress axis reverses dendritic cell suppression *in vivo*, which indicates that abnormal lipid metabolism leads to antigen presentation anergy.

Mitochondrial dysfunction adds a further layer to these metabolic abnormalities. Besides impaired oxidative phosphorylation in myeloid cells, lymphocyte mitochondrial metabolism is also affected. In a murine sepsis model, Reizine et al. (45) observed marked T cell mitochondrial dysfunction, with citrulline supplementation partially restoring mitochondrial function and T cell proliferation. Myeloid-derived oxidative stress can directly damage lymphocyte mitochondria. Reactive oxygen species (ROS) released by MDSCs can impair CD8-positive T cell mitochondrial function and weaken their metabolic fitness (2, 5). This mechanism links the abnormal expansion of innate immunity with the functional exhaustion of adaptive immunity, and shows that immune paralysis is not a simple sum of independent energy deficits across subpopulations.

While limiting cellular energy supply, metabolic abnormalities also stabilize the hyporesponsive phenotype through downstream epigenetic remodeling (4, 31). Domínguez-Andrés et al. (46) showed that the IRG1/ACOD1-itaconate pathway represents a central metabolic regulator linking innate immune tolerance and trained immunity, in part through modulation of succinate dehydrogenase activity and associated metabolic-epigenetic reprogramming. These changes can stabilize immune hyporesponsiveness at the transcriptional level. This process contributes to the persistence of immune paralysis. Unlike innate deactivation and lymphocyte apoptosis, which are early events, metabolic-epigenetic reprogramming is a slower, cumulative process. It may determine not whether immunosuppression occurs but whether it continues after pathogen clearance, which partly explains why immune paralysis can outlast the infection itself (4, 31, 46).

Stimuli such as β-glucan can induce enhancing epigenetic reprogramming (trained immunity), whereas repeated LPS stimulation drives suppressive reprogramming and endotoxin tolerance (4, 31, 46). Both act at the metabolic-epigenetic level to produce opposing functional outcomes. This directional tunability suggests that the metabolic and epigenetic axes may themselves become intervention targets. However, while these metabolic-epigenetic pathways provide a plausible mechanistic basis for immune paralysis, current evidence is derived predominantly from experimental systems and has not yet established these pathways as validated therapeutic targets in human sepsis.

### 3.4 Immune checkpoints and the sustained immunosuppressive network

The PD-1/PD-L1 axis is the most studied immune checkpoint pathway in sepsis, and clinical evidence for checkpoint pathways is more mature than for metabolic-epigenetic ones (40–42). In septic patients, PD-1 is upregulated on peripheral T cells and monocytes, and PD-L1 rises on monocytes, endothelial cells, and various parenchymal cells (6, 40, 42, 47). After PD-1 binds PD-L1, SHP-2-mediated inhibition attenuates downstream T cell receptor (TCR) signaling, which restricts T cell proliferation, lowers IL-2 production, and weakens effector function (40, 41).

Checkpoint molecule expression changes over the clinical course of sepsis (40, 42, 48). In one longitudinal study of patients with sepsis, PD-1 on CD4-positive T cells, CD8-positive T cells, and Tregs rose gradually after onset and peaked around two weeks (48). In the same study, PD-L1 on monocytes, neutrophils, and dendritic cells showed a biphasic pattern of early elevation, a transient decline on day 3, and a subsequent increase (48). Clinical studies show that increased PD-1/PD-L1 signaling is associated with impaired immune function and adverse outcomes during sepsis (40, 42). Soluble PD-L1 (sPD-L1) is also elevated in septic patients and has been associated with adverse clinical outcomes (42). These findings show that checkpoint upregulation, monocyte deactivation, and lymphocyte exhaustion can coexist during the sustained suppressive phase. The observed temporal patterns suggest that checkpoint upregulation may serve as a molecular readout of the maintenance phase of immune paralysis rather than its initial trigger, and that, together with metabolic-epigenetic locking, it may belong to the slower variables that govern how long suppression lasts.

Beyond PD-1/PD-L1, other inhibitory receptors such as TIM-3, LAG-3, CTLA-4, and BTLA are also implicated in sepsis-associated immune dysfunction (38, 41, 49). VISTA, a B7 family member, and the myeloid CD200/CD200R1 axis have recently been added to this suppressive network (37, 38, 41). Preclinical evidence suggests that these additional inhibitory pathways may contribute to T cell dysfunction and sustained immune suppression, although their therapeutic relevance in human sepsis remains uncertain (37, 38, 41).

### 3.5 Temporal stratification of the underlying mechanisms

Taken together, these four levels can be separated by time scale, and this separation connects directly to the recovery trajectories discussed in the following section. Innate deactivation and lymphocyte apoptosis are early, rapid events that appear within hours to days of onset and set the initial depth of immunosuppression (2, 6, 11, 36). Metabolic-epigenetic reprogramming and sustained checkpoint upregulation are slower, cumulative processes that may determine whether immunosuppression continues beyond the acute phase (4, 31, 40, 46, 48). The early events govern how deep immune paralysis becomes, and the slower variables influence how long it lasts. Whether a patient moves toward rapid recovery or sustained hyporesponsiveness is therefore set not by any single mechanism but by how these fast and slow variables combine at the individual level. This temporal stratification provides the mechanistic frame for the heterogeneity of immune recovery and serves as the starting point for the longitudinal view developed next.

## 4 Dynamic trajectories of immune paralysis: from single time points to longitudinal evolution

The immune response in sepsis can shift substantially within hours to days (7, 10, 13, 16). Fixed time-point assessment is easy to perform but cannot capture the direction, speed, and rhythm of this process. At any given moment, different patients may sit at different stages of the immune disease course (7, 16, 50). Building on the temporal stratification of mechanisms in the previous section, this section takes a longitudinal view of how immune paralysis evolves. The central question is no longer which suppressive mechanisms exist but how the combination of fast and slow variables unfolds over time into observable recovery trajectories. A growing body of work now characterizes these trajectories longitudinally and, on that basis, re-examines subtyping and prognostic frameworks built on single time points (11–16, 50).

### 4.1 Methodological evolution from cross-sectional endotypes to trajectory endotypes

Efforts to molecularly phenotype septic patients based on host immune heterogeneity began over the past decade (16, 50). Davenport et al. (51) proposed two endotypes, SRS1 and SRS2, through whole-blood gene expression analysis, with the former showing immunosuppressive features and correlating with higher mortality. Scicluna et al. identified four molecular endotypes, MARS1 through MARS4, in an independent cohort, which refined the immune heterogeneity landscape (16). By integrating 14 sepsis transcriptome datasets, Sweeney et al. (52) proposed three robust classifications, inflammopathic, adaptive, and coagulopathic, validated in another 9 datasets. More recent work has emphasized the need to reconcile these classifications across cohorts and to examine their temporal stability (50). These efforts established that biologically distinguishable subtypes exist in sepsis (16, 50–52). They largely rely on single-time-point sampling, however, and do not address the stability of endotypes over the disease course, which gave rise to the research focus on endotype trajectories (50, 53).

### 4.2 Limitations of single-time-point assessment

Immune phenotypes can shift substantially within hours to days of sepsis onset (7, 10, 16, 54). A hyperinflammatory state on day 1 of ICU admission may evolve into profound immunosuppression by day 3, while different patients may follow the reverse or other temporal patterns (2–4, 50). Against this rapid change, a single test result has very limited representativeness and reflects only an incidentally sampled transient snapshot on a continuous spectrum. Taking mHLA-DR as an example, the same absolute value on day 1 can carry different biological meaning depending on its position within the trajectory (11, 12, 14, 25). The value might mark the bottom of a rebound after a rapid drop, which suggests transient suppression, or an intermediate stage of a slow downward trend, which predicts continued deterioration.

There are also marked differences in when different patients enter immune paralysis (7, 16, 53). Cross-sectional comparison at the same time node can conflate patients at different stages of the immune disease course and obscure true biological signals. Through biomarker measurement every 8 hours in 345 septic patients, Rademaker et al. (53) found that approximately one-third of subgroup peers changed classification at each measurement interval. This transition reflects both genuine biological evolution and statistical classification uncertainty arising from measurement error and blurred clustering boundaries (53). This methodological limitation may partly contribute to difficulties in interventional trials based on single-time-point phenotyping, because the true immune status of enrolled patients may no longer match the assessment at the time of inclusion (28, 50, 53).

### 4.3 The first 1 to 2 weeks are the critical window for dynamic changes

Longitudinal studies indicate that the first 1 to 2 weeks after sepsis onset represent a particularly informative window for observing changes in immune status and divergence in recovery trajectories (11–16, 25, 50, 53, 55). The clinical importance of this window lies not only in the dynamic evolution of immune indices but also in its overlap with a clinically important period during which secondary and ICU-acquired infections frequently emerge (6, 8, 20).

Within this window, multiple immune biomarkers change continuously, but their recovery rhythms differ. A prospective study by Samuelsen et al. in 2024 (55) compared the dynamic changes of mHLA-DR, ALC, and ex vivo stimulated cytokine release in 64 critically ill patients (31 with sepsis, 33 non-sepsis) over the first two weeks after ICU admission. A significant rise in mHLA-DR was seen mainly in the non-sepsis subgroup, ALC recovery was more prominent in sepsis patients, and ex vivo cytokine production (TNF and IL-6) improved significantly over time in sepsis patients (55). This asynchronous recovery across biomarkers indicates that the immune system does not repair itself as a single synchronized whole. Instead, different functional modules rebuild independently at their own rhythms. A similar asynchrony appears at the level of checkpoint molecules. In one longitudinal study, PD-L1 followed a biphasic course after sepsis onset, with early elevation, a decline on day 3, and a subsequent increase (48). This rhythm both overlaps in time and diverges in direction from mHLA-DR.

The clinical implication of this asynchronous recovery is that a single indicator cannot fully reflect the longitudinal evolution of immune status during the window. In their study of 1,023 patients with septic shock, Monneret et al. (25) observed that serial assessment of mHLA-DR during the first ICU week provided more information about persistent immune dysfunction than an isolated early measurement. These findings support a multi-indicator, multi-time-point strategy for immune monitoring during the first 1 to 2 weeks, because baseline detection or a single indicator cannot accurately track the dynamic trajectory (23, 25, 27, 50, 55). From a mechanistic standpoint, this window is critical because it is the overlap period in which the early fast variables, innate deactivation and lymphocyte apoptosis, settle, while it is not yet clear whether the slow variables, metabolic-epigenetic locking and checkpoint upregulation, will engage. Trajectory divergence occurs here precisely because the slow variables that govern persistence reveal their direction during this period.

### 4.4 Different patients show different immune recovery trajectories

Existing longitudinal evidence supports two broad recovery patterns, including relatively rapid recovery and sustained hyporesponsiveness, while a third fluctuating or transitional pattern may be considered a hypothesis-generating category (11, 12, 14, 25, 50, 53). These patterns can be conceptually related to different combinations of the fast and slow variables described in the previous section.

Rapid recovery patients experience relatively brief immunosuppression, with major indices recovering during the early course, a lower risk of secondary infection, and generally favorable outcomes (12, 14, 25). Mechanistically, these patients are dominated by the early fast variables. Innate deactivation and lymphocyte apoptosis still occur, but suppressive metabolic-epigenetic reprogramming is not firmly locked in and checkpoint upregulation stays limited, so immune function recovers before the slow variables fully accumulate. This mechanistic interpretation should be regarded as a conceptual synthesis rather than a directly validated molecular trajectory.

Sustained hyporesponsive patients show persistently abnormal immunosuppression markers, which are associated with a higher incidence of secondary infection, prolonged organ dysfunction, and adverse outcomes (7, 12, 14, 20, 25). This phenotype may correspond to a slow-variable-dominated situation. Metabolic-epigenetic reprogramming, delayed and persistent expansion of M-MDSCs, and sustained upregulation of checkpoints such as PD-1 and PD-L1 may together contribute to maintaining immune paralysis beyond pathogen clearance and delaying recovery (34, 46, 48). This also explains why, for these patients, persistent rather than transient immunosuppression is the true target of intervention (27, 56).

In contrast to the empirically derived classifications described above, in some patients, immune status fluctuates repeatedly between suppression and relative activation, a pattern that can be labeled the proposed “fluctuating transition” trajectory. This proposed trajectory currently lacks direct validation as a discrete phenotype from large-sample trajectory clustering studies. Its mechanism is also unclear and may reflect a transitional state in which the fast and slow variables have not reached a stable balance. High-frequency longitudinal sampling nevertheless demonstrates frequent short-term transitions between biomarker-defined immune profiles (53). Until further longitudinal clustering evidence is available, this hypothesis-generating trajectory should be explicitly treated as a conceptual category, clearly distinguished from empirically derived recovery phenotypes.

Several longitudinal studies provide empirical support for the heterogeneity of recovery trajectories. In a 20-year cohort of 1,023 patients with septic shock, Monneret et al. (25) identified three trajectories by K-means clustering of mHLA-DR during the first ICU week: a sustained low-expression pattern, an improving pattern, and a high-expression pattern. The sustained low-expression pattern was associated with increased risk of ICU-acquired infection and adverse mortality outcomes. At higher temporal resolution, the high-density sampling by Rademaker et al. (53) showed that shifts in immune classification could occur within 48 hours of ICU admission, which points to a shorter time scale for state change than previously assumed. In a much larger population, a multi-cohort machine learning study by Zhang et al. (57) covering 24,450 septic adults identified three patterns based on 28-day SOFA trajectories: rapid recovery (42.3%), slow recovery (35.8%), and clinical deterioration (21.9%). These SOFA-based patterns reflect trajectories of clinical organ dysfunction rather than direct immune phenotypes, but they provide complementary evidence for marked heterogeneity in recovery after sepsis.

Although these studies differ in population scale and assessment metrics, they agree on the core conclusion. Immune and clinical recovery trajectories are markedly heterogeneous, and persistent dysfunction represents an important adverse recovery pattern (25, 50, 53, 57).

Assessment at a single time point cannot reflect the speed, direction, and rhythm of immune recovery. The recently proposed concept of endotype trajectories was introduced against this background, and emphasizes that understanding of sepsis-induced immune paralysis must extend from static classification to evolution along the temporal dimension (50, 53). This evolution over time is not random. It is driven by differences in time scale among the mechanisms set out in the previous section, which is the logical thread that joins the mechanistic and trajectory discussions into one argument.

## 5 Main monitoring indicators of sepsis-induced immune paralysis

### 5.1 Monocyte HLA-DR

Downregulation of human leukocyte antigen-DR expression on circulating monocytes (mHLA-DR) is one of the most extensively studied biomarkers of innate immune dysfunction in sepsis (5, 22) HLA-DR is essential for antigen presentation and reflects the functional state of circulating monocytes. During sepsis-associated immunosuppression, reduced mHLA-DR expression is associated with impaired antigen-presenting capacity and monocyte deactivation (11, 21). Accordingly, mHLA-DR is best regarded primarily as an immune-phenotyping and prognostic biomarker, although it has also been used as a pharmacodynamic marker in studies evaluating immune-restorative interventions (11, 12, 14, 18, 23).

Clinically, mHLA-DR is most commonly quantified by standardized flow cytometry and may be reported as antibodies bound per cell (AB/C) or receptors per cell. Several numerical cutoffs have been proposed, but these thresholds should be interpreted within the specific analytical and clinical contexts in which they were derived. Values below approximately 15,000 AB/C have been associated with more pronounced immune dysfunction or greater disease severity in some adult cohorts, whereas thresholds around 8,000 AB/C have been used in selected studies to operationalize marked monocyte deactivation or severe immunosuppression (24, 25). these values do not constitute universally accepted definitions of sepsis-induced immunoparalysis. Reported cutoffs vary according to assay methodology, calibration procedure, flow-cytometry platform, patient population, and timing of measurement, and thresholds derived from different studies should therefore not be considered directly interchangeable (23, 26, 27).

Beyond the absolute value at a single time point, the longitudinal trajectory of mHLA-DR provides important prognostic information. Persistently low mHLA-DR expression or failure of recovery during the first several days after sepsis onset has been associated with secondary or ICU-acquired infection and increased mortality, whereas gradual recovery is generally associated with more favorable outcomes (12, 14, 30). These findings suggest that serial measurements, particularly during the early days of critical illness, may better characterize persistent monocyte dysfunction than an isolated baseline measurement. However, an association with subsequent clinical outcomes should not be interpreted as evidence that mHLA-DR alone can predict benefit from a specific immunostimulatory treatment.

More recently, the ImmunoSep randomized clinical trial provided early proof-of-concept for incorporating mHLA-DR into biomarker-enriched treatment stratification (28). Within the trial-specific classification framework, sepsis-induced immunoparalysis was defined in part by fewer than 5,000 HLA-DR receptors on CD45/CD14 monocytes, and patients assigned to this immune phenotype received recombinant human interferon-γ. This threshold should therefore be interpreted as a protocol-specific treatment-stratification criterion rather than a universal diagnostic cutoff for immunoparalysis. Although the trial demonstrated the feasibility of linking biological stratification to targeted immunotherapy and reported improvement in its primary organ-function endpoint, it did not establish a definitive mortality benefit. Thus, current evidence supports mHLA-DR primarily for immune phenotyping, prognosis, and pharmacodynamic monitoring, while its role as a validated predictive biomarker for treatment response remains to be confirmed in larger prospective trials.

Despite its biological and prognostic relevance, routine implementation of mHLA-DR remains constrained by several technical factors. Accurate measurement requires standardized flow-cytometric protocols, appropriate calibration, trained laboratory personnel, and timely processing of blood samples. Inter-laboratory variability and differences among analytical platforms further limit the direct transferability of numerical thresholds across centers (26). Therefore, future clinical translation will require greater methodological harmonization, repeated rather than isolated assessment, and prospective validation of both prognostic and treatment-selection thresholds.

### 5.2 Absolute lymphocyte count and persistent lymphopenia

Compared with more complex flow-cytometric assays, absolute lymphocyte count (ALC) is a readily available, simple, and low-cost marker for assessing the degree of circulating lymphocyte depletion during sepsis. Excessive inflammatory signaling and stimulation by pathogen-associated molecular patterns (PAMPs) during early sepsis can induce substantial apoptosis of lymphocytes, including CD4+ T cells, CD8+ T cells, and B cells (2, 6, 35). Although transient lymphopenia is common during the acute phase, persistent lymphopenia has been associated with sustained adaptive immune dysfunction and adverse clinical outcomes. In several adult cohorts, failure of ALC to recover above approximately 1.0–1.2 × 10^9^/L by ICU day 4 or later has been used to characterize persistent lymphopenia (15, 23, 39). However, these thresholds are cohort- and time-dependent and should not be interpreted as universal diagnostic cutoffs for immunoparalysis. Importantly, a reduced ALC reflects the quantity of circulating lymphocytes but does not directly measure their functional state; therefore, persistent lymphopenia alone does not establish functional T-cell exhaustion. Current evidence supports ALC primarily as an accessible phenotyping and prognostic marker, whereas its ability to predict response to immune-restorative therapy remains unestablished. Persistent lymphopenia has nevertheless been associated with increased mortality and susceptibility to secondary or opportunistic infections (15, 23, 39).

### 5.3 Cytokine release capacity after *ex vivo* blood stimulation

In contrast to static measurements of circulating cytokines, ex vivo functional assays of peripheral blood provide a much truer reflection of the immune effector cells’ actual “reactivity” when faced with a secondary hit (31, 58). The ex vivo lipopolysaccharide (LPS) stimulation test serves as the classic functional model for unmasking “endotoxin tolerance” (23, 31). During the immunoparalysis phase of sepsis, the capacity of monocytes to release pro-inflammatory mediators (e.g., TNF-α) upon rigorous external stimulation drops precipitously (11, 32).This functional “paralysis” of the immune system significantly outperforms static biochemical concentrations in predicting secondary infections and patient mortality (2, 5, 58, 59).

### 5.4 Cytokines and the inflammatory/anti-inflammatory balance

Systemic cytokine storms and the subsequent anti-inflammatory cascades (e.g., massive IL-10 release) constitute a complex network of immune dysregulation (60, 61). Studies have shown that an abnormally high IL-6/IL-10 ratio is closely linked to deeper immunosuppression (62, 63).This phenomenon vividly illustrates the paradox model of sepsis: an early inflammatory defense mechanism intended to protect the host is rapidly inverted into a “double-edged sword” following overactivation, ultimately explaining clinical failures and persistent immune exhaustion even after successful pathogen clearance (61, 64). Because cytokines possess extremely short half-lives and are drastically influenced by the disease time window, their specificity is limited; thus, they must be utilized as a combined panel for longitudinal, multi-dimensional assessment (60, 65).

### 5.5 Immune checkpoints and composite immune biomarkers

With deeper insights into the mechanisms of sepsis-induced T-cell exhaustion, co-inhibitory immune checkpoint molecules, particularly PD-1/PD-L1, have emerged as cutting-edge monitoring targets (40, 66). The upregulation of PD-1 and PD-L1 on T cells and monocytes not only directly undermines immune surveillance and bactericidal capacity but is also highly associated with extremely poor prognoses (41). Given the immense heterogeneity of clinical phenotypes, solitary biomarkers are often insufficient, making the construction of composite immune marker models an inevitable trend (47, 66). For instance, combining PD-1 (reflecting adaptive immune exhaustion), cluster of differentiation 64 (CD64, indicating innate immune activation), and HLA-DR (representing antigen presentation) establishes a precise, multidimensional immune profile. This significantly enhances the accuracy of stratifying critically ill patients and evaluating potential therapeutic benefits (5, 42) The core definition, clinical threshold, functional pros and cons of representative immune monitoring biomarkers are systematically summarized in **Table 1**.

**Table 1:** Summary of key immune monitoring markers for sepsis-induced immunoparalysis.

Marker	Core Definition	Clinical Threshold/Cutoff	Primary Role	Advantages	Limitations	Reference
Monocyte HLA-DR (mHLA-DR)	HLA-DR expression level on monocytes, measured by flow cytometry;	<15,000AB/C: sepsis severity <8,000 AB/C: severe immunoparalysis <5,000 receptors/cell: ImmunoSep trial SII criterion;	Gold standard for innate immune deactivation; predict secondary infection and mortality;	High prognostic value; directly reflect antigen-presenting function	Requires flow cytometry; strict sample processing time; poor cross-center standardization	(12, 29, 50, 67)
Absolute Lymphocyte Count (ALC)	Peripheral blood lymphocyte count;	Persistent lymphopenia: ALC <1.0–1.2×10^9^/L (ICU day 4+);	Assess adaptive immune exhaustion; predict long-term mortality and opportunistic infections;	Easy to detect; low cost; high clinical accessibility	Non-specific; cannot reflect lymphocyte function	(15, 23, 39)
Exvivo cytokine production capacity	Pro-inflammatory cytokine release (TNF-α, IL-6, IL-12) after LPS stimulation;	Markedly reduced release compared with healthy controls;	Unmask endotoxin tolerance; evaluate immune cell reactivity;	Better predict secondary infection than static cytokines	Time-consuming; labor-intensive; not suitable for rapid bedside test	(11, 32, 58)
Systemic cytokines (IL-6, IL-10, ratio)	Circulating pro-/anti-inflammatory cytokine concentration;	High IL-6/IL-10 ratio: deep immunosuppression;	Reflect inflammatory/anti-inflammatory imbalance; assist severity assessment;	Easy to detect; widely used in clinical practice	Short half-life; affected by disease time window; low specificity	(60–62)
Immune checkpoints (PD-1/PD-L1, TIM-3, LAG-3)	Expression of inhibitory receptors on T cells/monocytes/soluble form;	Progressive upregulation after sepsis onset; PD-L1 biphasic change;	Reflect T-cell exhaustion; predict poor prognosis;	Novel target; correlate with immune paralysis severity	Requires flow cytometry; lack unified clinical cutoff; limited clinical validation;	(40, 41, 66)
Composite immune biomarkers	Panelcombining mHLA-DR, ALC, PD-1, CD64, etc.	Integrated scoring based on multiple markers	Comprehensive immune phenotype stratification; improve prediction accuracy	Overcome single-marker limitation; higher precision	Complex calculation; nlack unified scoring system; difficult for clinical promotion	(5, 42, 66)

AB/C, antibodies bound per cell; SII, sepsis-induced immunoparalysis.

All thresholds and evaluations are derived from Sections 5.1–5.5 and key clinical studies including the ImmunoSep trial.

This table summarizes the core biomarkers for monitoring sepsis-induced immunoparalysis, including clinical cutoffs, strengths and limitation

## 6 Monitoring strategies for sepsis-induced immune paralysis

### 6.1 From single indicators to combined monitoring and transcriptomic panels

Historically, the assessment of immune dysfunction in sepsis relied on isolated biomarkers. However, given the profound heterogeneity of the host response, relying on a single indicator is mathematically and biologically insufficient to capture the full spectrum of immune dysregulation (2, 4, 5). The current paradigm is rapidly shifting toward a comprehensive, multidimensional framework (66). Future monitoring strategies must transition to a combined approach that integrates monocyte HLA-DR (mHLA-DR) expression, ALC, ex vivo functional assays (such as endotoxin stimulation), and clinical severity (15, 42, 58, 68) Crucially, to deepen our assessment beyond cellular phenotypes, this strategy must construct composite immune indices. For instance, integrating standardized ex vivo whole-blood stimulation assays (e.g., the TruCulture^®^ system) can precisely unmask profound endotoxin tolerance (69). Furthermore, integrating these functional readouts with rapid transcriptomic endotyping provides a high-resolution map of the host’s immunological state, significantly improving the stratification of patients at high risk for secondary infections and mortality compared to traditional markers alone (42, 60, 70).

### 6.2 From a single test to repeated dynamic assessments

Sepsis is a rapidly evolving condition; thus, a single baseline measurement fails to identify patients who develop protracted immunosuppression (23, 30). A longitudinal monitoring axis is biologically essential (71, 72). Assessments should follow a precise timeline: a baseline evaluation upon ICU admission or diagnosis, followed by an early re-evaluation at 24 to 48 hours to gauge the initial immune trajectory (36, 64). Crucial risk re-stratification must occur between days 3 and 5, a critical window where the failure of mHLA-DR or ALC to recover strongly correlates with adverse outcomes (32, 73). Finally, assessments beyond day 7 are vital to differentiate between immune recovery and a state of persistent, late-phase immunoparalysis (31, 74). To mathematically decipher these complex longitudinal changes, assessments must shift from observational tracking to sophisticated latent class trajectory modeling (LCTM). Tracking these data through LCTM can identify patients locked in a persistently deteriorating trajectory, a phenotype that robustly correlates with the transition into the persistent inflammation, immunosuppression, and catabolism Syndrome (PICS)—a maladaptive state responsible for the majority of chronic critical illness and late sepsis mortality (75).

### 6.3 Overcoming analytical bottlenecks: standardization and microfluidic POC

The translation of immune monitoring from the research bench to the bedside faces significant pragmatic hurdles (11, 59) Flow cytometry—the gold standard for quantifying mHLA-DR and immune checkpoints—demands highly trained personnel, specialized equipment, and strict sample handling times, leading to a lack of standardization across different centers (62, 76). To achieve bedside utility, the field must aggressively adopt standardized quantification protocols, such as utilizing molecules of equivalent soluble fluorochrome (MESF) calibration beads to transition mHLA-DR from arbitrary mean fluorescence intensity to highly reproducible absolute value (26) Furthermore, functional ex vivo assays are often time-consuming and labor-intensive (36, 63). To overcome these barriers, the field must aggressively pursue point-of-care (POC) solutions. The development of rapid, fully automated microfluidic cartridge systems capable of returning host-transcriptome scores or measuring immune checkpoints from whole blood within hours is effectively bypassing the logistical bottlenecks of centralized laboratories (77) These simplified immune scoring systems and bedside platforms are crucial to delivering real-time, actionable data to clinicians (1, 78).

### 6.4 Actionable clinical integration: the “dual-axis” assessment and immuno-SOFA

Crucially, immunological monitoring should never be interpreted in a vacuum, divorced from the patient’s overarching clinical context (40). To bridge the gap between immunological biomarkers and widely used clinical scoring systems, we propose a conceptual “dual-axis assessment” framework that integrates dynamic immune trajectories with the Sequential Organ Failure Assessment (SOFA) score. Traditionally, the SOFA score quantifies the macro-level severity of organ dysfunction, whereas immune markers—such as mHLA-DR and absolute lymphocyte count (ALC)—provide complementary information on the host immune state (58). Evaluating these two dimensions together may provide a structured approach to contextualizing immune abnormalities alongside the clinical trajectory. Importantly, this dual-axis framework should currently be regarded as a hypothesis-generating proposal rather than a validated clinical score or treatment algorithm.

Within this framework, a patient with a persistently high SOFA score but relatively preserved immune indices may represent a different biological phenotype from a patient with simultaneous organ dysfunction and persistent immune abnormalities. For example, persistently depressed mHLA-DR together with an unfavorable SOFA trajectory may identify a high-risk immune-clinical phenotype warranting further investigation. However, such a combination should not be assumed to establish the cause of ongoing organ failure or to define eligibility for immunostimulatory therapy. In particular, thresholds such as mHLA-DR <5,000 receptors/cell are derived from specific study frameworks, including ImmunoSep, and should not be interpreted as universally validated treatment-selection criteria.

Moving forward, an “Immuno-SOFA” composite framework could be explored as a research tool integrating organ dysfunction with longitudinal immune measurements. Conceptually, a future composite score could combine SOFA with standardized immune variables, for example Ct=αSOFAt+k∑wkzk,t where z_{k,t} represents an immune marker measured at time t, and w_k represents its relative contribution. No weighting scheme is currently validated, and future weights should be derived from appropriately designed development cohorts rather than fixed *a priori*. Such models would also require prespecified approaches to missing longitudinal measurements, internal and external validation, and evaluation against clinically relevant outcomes such as secondary infection, persistent organ dysfunction, and short- and longer-term mortality. More advanced computational approaches, including reinforcement learning, remain speculative and are therefore considered separately in the Future Directions section.

## 7 Immunoparalysis-guided precision intervention

### 7.1 The immunological rationale for paralysis-directed therapy

The history of immunoadjuvant therapy in sepsis is littered with negative trials, largely because patients were enrolled without *a priori* evidence of an immunosuppressed phenotype (37). Agents such as GM-CSF or IFN-γ consistently failed to improve survival in unselected sepsis populations, obscuring the possibility that they could be effective—or even lifesaving—in the biologically appropriate subgroup (79). The central therapeutic hypothesis has accordingly shifted from “treat all” to “identify the hyporesponsive host and restore immune competence.” Realizing this paradigm demands that a plausible pharmacodynamic mechanism—such as monocyte reactivation or T-cell restoration—be explicitly linked to a measurable pre-treatment deficit (e.g., low mHLA-DR, persistent lymphopenia, or blunted ex vivo cytokine release) and, ultimately, to patient-centered outcomes (80). Importantly, this framework requires distinguishing between prognostic biomarkers, which indicate risk of adverse outcomes regardless of treatment, and predictive biomarkers, which identify patients likely to benefit from a specific intervention—a distinction that remains insufficiently characterized for most immune markers in sepsis.

### 7.2 Biomarker-guided precision immunotherapy: the ImmunoSep trial and beyond

The ImmunoSep randomized trial, published in JAMA, represents an important proof-of-concept study rather than definitive clinical evidence (28). The trial employed dual-biomarker stratification, classifying patients as having either macrophage-activation-like syndrome or sepsis-induced immunoparalysis, with the latter defined, in part, by a CD45/CD14 monocyte HLA-DR density <5000 antibodies per cell. Immunoparalysed patients received recombinant human IFN-γ, and the study reported significant improvement in organ function at day 9. The sample size was moderate; the primary endpoint was organ function rather than mortality; the study was not designed to establish long-term patient-centered outcomes; and the biomarker threshold and treatment effect require replication in broader, heterogeneous populations. In particular, a survival benefit remains unproven, and the generalizability of the stratification approach beyond the trial setting is unknown. Therefore, ImmunoSep provides proof of concept that bedside biological stratification is feasible and can indeed modify intermediate endpoints, but it does not yet establish biomarker-guided immunostimulation as a standard of care.

Equally instructive are the lessons for future trial design. First, the timing of biomarker measurement must align with the expected window of reversibility. As a recent editorial articulated, immune stimulation in sepsis should be guided by careful attention to timing, stratification, and monitoring, and only persistent—rather than transient—immunosuppression justifies therapeutic intervention; administering an immune stimulant after irreversible organ injury has set in is unlikely to confer benefit (56). Second, a single marked such as mHLA-DR alone may be insufficient, this concern is substantiated by a 20-year cohort study of over 1,000 septic shock patients, which demonstrated that a single early mHLA-DR measurement cannot reliably identify persistent immunosuppression and that dynamic tracking over the first week is essential (27). Moreover, the fact that low mHLA-DR predicts poor outcome does not necessarily imply that it can identify patients who will respond to immunostimulation; its role as a prognostic biomarker is more established than its role as a predictive biomarker. Future trials may therefore need to adopt composite immunological scores that incorporate absolute lymphocyte count, ex vivo stimulation data, and immune checkpoint expression, and possibly treatment-specific predictive markers, to distinguish risk from likely treatment benefit.

### 7.3 Candidate immune-enhancing strategies under monitored guidance

Once a trajectory of persistent immunoparalysis is confirmed, several therapeutic strategies merit consideration, although their evidence bases differ substantially and none has yet been shown to improve mortality in adequately powered randomized trials.

First, recombinant human IFN-γ aims to reverse monocyte deactivation and upregulate HLA-DR, thereby restoring antigen-presenting capacity; supportive evidence comes primarily from small proof-of-concept studies and the recent ImmunoSep trial, which used organ function as an endpoint (11, 28, 81). In parallel, GM-CSF promotes myeloid cell maturation and function; evidence derives mainly from small randomized trials in selected patients with confirmed monocyte deactivation, showing improved mHLA-DR levels and reduced secondary infection rates, but not yet mortality benefit (18, 82). Another promising approach targets the lymphoid compartment. Interleukin-7 (IL-7, CYT107), a T-cell homeostatic cytokine, expands naïve and memory T cells and has been evaluated in early-phase studies in sepsis patients with persistent lymphopenia, yielding T-cell expansion with an acceptable short-term safety profile; long-term safety and efficacy remain unknown (83). In parallel, immune checkpoint inhibitors—in particular, anti-PD-1/PD-L1 antibodies—are entering early-phase sepsis trials, driven by the observation that PD-1 expression on T cells is elevated during prolonged immunosuppression. However, these agents carry a particular risk of precipitating hyperinflammation, and their safety margin in sepsis remains poorly defined; therefore, patient selection must be exceptionally careful, and current evidence is limited to phase 1/2 settings (49).

Importantly, in all these strategies, the intervention should be conceptualized not as a “rescue” for end-stage patients but as a timed therapy delivered during a specific immunological window. A systematic summary of the evidence maturity, known safety concerns, and unresolved questions for each candidate therapy is presented in **Table 2**.

**Table 2:** Evidence maturity, safety considerations, and unresolved questions for candidate immune-enhancing strategies in sepsis-induced immunoparalysis.

Therapy	Evidence maturity	Known safety concerns	Unresolved questions
IFN-γ	Phase 2 RCT (ImmunoSep) using organ function as endpoint; earlier small proof-of-concept studies	Flu-like symptoms; theoretical risk of exacerbating hyperinflammation if given in MALS phenotype	Mortality benefit unproven; optimal dose and timing unclear
GM-CSF	Small RCTs in selected patients with monocyte deactivation; no mortality benefit yet	Injection site reactions; possible pulmonary toxicity; risk of worsening inflammation	Which patients benefit most; long-term safety
IL-7 (CYT107)	Phase 1/2 studies in sepsis with persistent lymphopenia; T-cell expansion shown	Short-term acceptable; long-term safety unknown	Efficacy for clinical outcomes; optimal dosing window
Anti-PD-1/PD-L1	Early-phase trials (Phase 1b) only	Hyperinflammation; immune-related adverse events; narrow therapeutic window	Safety margin in sepsis; identification of predictive markers

### 7.4 From “diagnosing immunoparalysis” to “matching the therapeutic window”

A central argument of this review is that the critical future step is not simply diagnosing immunoparalysis, but answering three temporal questions: *Is the host currently in a hyporesponsive state now? Is this state sustained or self-resolving? And is this a point at which intervention can still be effective?* The concept of a dynamic “treatment-eligible window” offers greater clinical value than a static diagnosis (38).

Translating this concept into practice requires a structured, stepwise decision framework. We therefore propose a hypothesis-generating three-stage algorithm that requires prospective validation before it can be considered a clinical recommendation. Stage 1 (Screening)—upon ICU admission, baseline immune profiling (mHLA-DR, absolute lymphocyte count) could be performed concurrently with standard clinical severity assessment (e.g., SOFA score). Stage 2 (Confirmation)—at 48–72 hours, repeated immune monitoring would be conducted to distinguish transient suppression from persistent immunoparalysis. Patients whose mHLA-DR remains <5,000 antibodies per cell and whose SOFA score shows no improvement or deterioration might be considered as potential candidates for immune restoration in the context of a clinical trial; however, this threshold is derived primarily from the ImmunoSep stratification framework and should not be interpreted as a universally validated definition of immunoparalysis (38, 55). Stage 3 (Intervention matching)—for patients meeting these provisional criteria, an immune-enhancing strategy (e.g., IFN-γ or GM-CSF) could be evaluated, with treatment response monitored by serial mHLA-DR measurement and organ function trajectories at day 5–7 (28). This proposed algorithm directly integrates immunological biomarkers with the Sequential Organ Failure Assessment (SOFA) score, a clinical tool already embedded in routine ICU practice, and is intended only as a conceptual bridge between research-grade immune monitoring and bedside decision-making, pending rigorous validation.

Making this judgment will require integrating repeated biomarker monitoring with the patient’s clinical trajectory—including persistent organ dysfunction, unresolved infection, and new nosocomial infection—and anchoring immune-directed decisions to established clinical scoring systems (55). Building on this concept, a proposed intervention framework for sepsis-related immunoparalysis is outlined in **Figure 2**, explicitly labelled as a hypothesis-generating approach requiring prospective validation.

**Figure 2:**
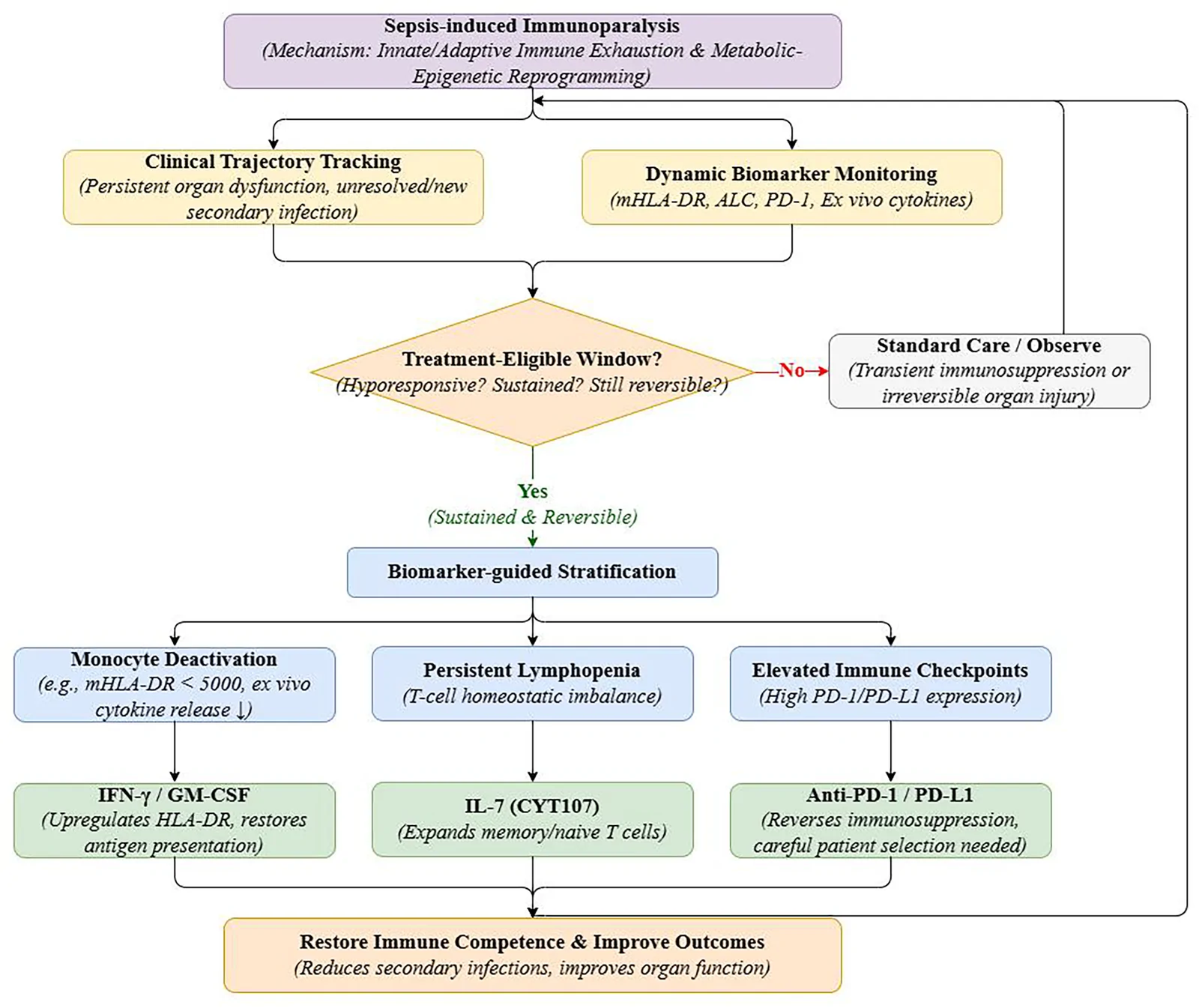
Proposed clinical algorithm for biomarker-guided precision immunotherapy in sepsis-induced immunoparalysis.

## 8 Current challenges and future perspectives

### 8.1 Lack of unified definitions and thresholds

Despite decades of investigation, no consensus exists on the precise thresholds that define immunoparalysis. Criteria for mHLA-DR (e.g., <5000 or <8000 antibodies/cell), absolute lymphocyte count (<0.5×10^9^/L or <1.0×10^9^/L), and ex vivo TNF-α release capacity vary substantially across studies (4). It is important to recognize that thresholds are assay- and platform-dependent, may differ between cohorts, are derived mainly from adult populations, and depend critically on the timing of measurement. In particular, the <5000 antibodies/cell threshold is closely tied to the ImmunoSep trial’s stratification framework and should not be regarded as a universal definition of immunoparalysis. This heterogeneity hampers cross-study comparison and impedes meta-analytic effort. A consensus-driven standardization initiative, perhaps analogous to the Sepsis-3 definitions, is urgently needed for immunological phenotypes in sepsis.

### 8.2 Dynamic trajectory research lacks standardization

The study of longitudinal immune trajectories is conceptually compelling yet methodologically fragmented. Studies differ markedly in sampling frequency—ranging from every 8 hours to daily or only on days 1, 3, and 7—as well as in observation duration, statistical approaches to trajectory clustering, and the availability of external validation cohorts. This variability makes it difficult to assess the generalizability of individual findings. Given emerging evidence that ICU immune classifications can shift substantially within 48 hours, single-timepoint stratification is increasingly untenable. Yet the field has not yet converged on a minimal standard for dynamic monitoring (84).

### 8.3 The gap from research-grade stratification to bedside implementation

Current immune profiling platforms—including standardized flow cytometry for mHLA-DR, multiplex cytokine panels, and ex vivo whole-blood stimulation—are operator-intensive, costly, and burdened by turnaround times that are incompatible with real-time clinical decision-making in most centers (27). Even when a research team succeeds in generating a trajectory-based immunological readout within 36 hours, the clinician at the bedside managing a deteriorating patient requires actionable information in a matter of hours. Bridging this gap will demand investment in rapid, automated platforms, simplified immune scoring systems, and point-of-care adaptations of functional assays. Critically, future immune monitoring tools must be designed for seamless integration with existing clinical workflow—for instance, by embedding immune parameters (e.g., a composite “immune-SOFA” subscore) into electronic health record dashboards that already display SOFA, APACHE II, or other severity scores. One such possibility is a conceptual “immune-SOFA” subscore, the construction, weighting of variables, handling of missing longitudinal data, and external validation of which remain entirely to be defined. Without such integration and validation, even the most accurate immunological readout risks remaining a research curiosity rather than a clinical decision tool.

### 8.4 Future directions

We anticipate four key advances that could unfold over the coming decade.

First, multi-marker composite immune scoring could distill mHLA-DR, absolute lymphocyte count, functional readouts, and clinical severity into a single clinically interpretable score—one that is explicitly anchored to established scoring systems such as SOFA to facilitate adoption at the bedside, although the methodology for such integration remains to be developed (85). Building on this, trajectory-based classification—rather than status-based labelling—would employ machine-learning algorithms that draw on repeated measurements to assign patients to dynamic immune trajectories, moving beyond single-timepoint categories. Furthermore, machine-learning-assisted immune stratification could integrate electronic health record data with streaming biomarker data to predict trajectory membership early and trigger timely, personalized interventions (86). Critically, any such interventions should be proposed within a time window defined not only by immunological parameters but also by clinical trajectory, including SOFA score dynamics, the course of organ dysfunction, and the emergence of secondary infections—thereby ensuring that the decision to intervene is both immunologically informed and clinically contextualized. Ultimately, these elements may converge into integrated monitor–stratify–intervene loops: closed-loop systems in which immunological monitoring directly informs precision immunotherapy delivery, and which would be rigorously tested within adaptive platform trials that embed biomarker-based subgroup analyses into the trial architecture from the outset.

## 9 Conclusion

In summary, sepsis-induced immunoparalysis is better understood as a heterogeneous and dynamically evolving immune state than as a uniform late-stage phenomenon. Repeated assessment using complementary immune markers may provide a more informative characterization of immune trajectories than isolated single-time-point measurements. However, most currently available biomarkers primarily provide phenotyping or prognostic information, whereas evidence supporting their use as predictive markers for treatment response remains limited. The ImmunoSep trial provides encouraging proof-of-concept for biomarker-enriched immunotherapy, but treatment-selection thresholds, composite frameworks such as Immuno-SOFA, and phenotype-matched intervention algorithms remain investigational. Future studies should prioritize prospective external validation, patient-important outcomes, and systematic safety assessment before these strategies are incorporated into routine clinical practice.

## Glossary

AB/C: antibodies per cell
ACOD1: aconitate decarboxylase 1
AI: artificial intelligence
ALC: absolute lymphocyte counts
BTLA: B and T lymphocyte attenuator
CD14: cluster of differentiation 14⁺
CD200: Cluster of Differentiation 200
CD200R1: Cluster of Differentiation 200 Receptor 1
CD200Rhigh: CD200 receptor-high
CD64: cluster of differentiation 64
CD80: costimulatory molecules cluster of differentiation 80
CD86: cluster of differentiation 86
CMV: cytomegalovirus
CTLA-4: cytotoxic T-lymphocyte-associated protein 4
DC: dendritic cell
EBV: Epstein–Barr virus
EHR: electronic health record
ER: endoplasmic reticulum
GM-CSF: granulocyte-macrophage colony-stimulating factor
H3K27ac: acetylation of histone H3 at lysine 27
H3K27me3: trimethylation of histone H3 at lysine 27
H3K4me3: trimethylation of histone H3 at lysine 4
HIF-1α: hypoxia-inducible factor-1 alpha
HLA-DRlow: low expression of human leukocyte antigen-DR
ICU: intensive care unit
IFN-γ: interferon-γ
IL-10: interleukin-10
IL-12: interleukin-12
IL-1β: interleukin-1 beta
IL-6: interleukin-6
IL-7: CYT107, Interleukin-7
IRG1: immune responsive gene 1
LAG-3: lymphocyte-activation gene 3
LCTM: latent class trajectory modelling
LPS: lipopolysaccharide
M1: the classically activated macrophages
MARS1: molecular diagnosis and risk stratification of sepsis endotype 1
MARS4: molecular diagnosis and risk stratification of sepsis endotype 4
MDSCs: myeloid-derived suppressor cells
MESF: molecules of equivalent soluble fluorochrome
mHLA-DR: monocyte human leukocyte antigen-DR
M-MDSCs: Monocytic myeloid-derived suppressor cells
NK: natural killer
PAMPs: pathogen-associated molecular patterns
PD-1: programmed death-1
PD-L1: programmed death-ligand 1
PICS: persistent inflammation, immunosuppression, and catabolism Syndrome
POC: point-of-care
RL: reinforcement learning
ROS: Reactive oxygen species
SDH: succinate dehydrogenase
SII: sepsis-induced immunoparalysis
SOFA: sequential organ failure assessment
sPD-L1: Soluble PD-L1
SREBF1: sterol regulatory element-binding transcription factor 1
TCR: T cell receptor
TET2: ten-eleven translocation 2
TGF-β: transforming growth factor beta
TIM-3: T cell immunoglobulin and mucin domain-containing protein 3
TNF: tumor necrosis factor
TNF-α: tumor necrosis factor-α
Tregs: regulatory T cells
VISTA: V-domain Ig suppressor of T cell activation

## References

[1] Singer M Deutschman CS Seymour CW Shankar-Hari M Annane D Bauer M . The third international consensus definitions for sepsis and septic shock (Sepsis-3). Jama. (2016) 315:801–10. doi: 10.1001/jama.2016.0287 26903338 PMC4968574

[2] Hotchkiss RS Monneret G Payen D . Sepsis-induced immunosuppression: from cellular dysfunctions to immunotherapy. Nat Rev Immunol. (2013) 13:862–74. doi: 10.1038/nri3552 24232462 PMC4077177

[3] Delano MJ Ward PA . The immune system's role in sepsis progression, resolution, and long-term outcome. Immunol Rev. (2016) 274:330–53. doi: 10.1111/imr.12499 27782333 PMC5111634

[4] van der Poll T van de Veerdonk FL Scicluna BP Netea MG . The immunopathology of sepsis and potential therapeutic targets. Nat Rev Immunol. (2017) 17:407–20. doi: 10.1038/nri.2017.36 28436424

[5] Venet F Monneret G . Advances in the understanding and treatment of sepsis-induced immunosuppression. Nat Rev Nephrol. (2018) 14:121–37. doi: 10.1038/nrneph.2017.165 29225343

[6] Boomer JS To K Chang KC Takasu O Osborne DF Walton AH . Immunosuppression in patients who die of sepsis and multiple organ failure. Jama. (2011) 306:2594–605. doi: 10.1001/jama.2011.1829 22187279 PMC3361243

[7] Shankar-Hari M Datta D Wilson J Assi V Stephen J Weir CJ . Early PREdiction of sepsis using leukocyte surface biomarkers: the ExPRES-sepsis cohort study. Intensive Care Med. (2018) 44:1836–48. doi: 10.1007/s00134-018-5389-0 30291379

[8] Prescott HC Angus DC . Enhancing recovery from sepsis: a review. Jama. (2018) 319:62–75. doi: 10.1001/jama.2017.17687 29297082 PMC5839473

[9] Iwashyna TJ Ely EW Smith DM Langa KM . Long-term cognitive impairment and functional disability among survivors of severe sepsis. Jama. (2010) 304:1787–94. doi: 10.1001/jama.2010.1553 20978258 PMC3345288

[10] Conway Morris A Anderson N Brittan M Wilkinson TS McAuley DF Antonelli J . Combined dysfunctions of immune cells predict nosocomial infection in critically ill patients. Br J Anaesth. (2013) 111:778–87. doi: 10.1093/bja/aet205 23756248

[11] Döcke WD Randow F Syrbe U Krausch D Asadullah K Reinke P . Monocyte deactivation in septic patients: restoration by IFN-gamma treatment. Nat Med. (1997) 3:678–81. doi: 10.1038/nm0697-678 9176497

[12] Monneret G Lepape A Voirin N Bohé J Venet F Debard AL . Persisting low monocyte human leukocyte antigen-DR expression predicts mortality in septic shock. Intensive Care Med. (2006) 32:1175–83. doi: 10.1007/s00134-006-0204-8 16741700

[13] Cajander S Rasmussen G Tina E Magnuson A Söderquist B Källman J . Dynamics of monocytic HLA-DR expression differs between bacterial etiologies during the course of bloodstream infection. PloS One. (2018) 13:e0192883. doi: 10.1371/journal.pone.0192883 29466395 PMC5821339

[14] Lukaszewicz AC Grienay M Resche-Rigon M Pirracchio R Faivre V Boval B . Monocytic HLA-DR expression in intensive care patients: interest for prognosis and secondary infection prediction. Crit Care Med. (2009) 37:2746–52. doi: 10.1097/CCM.0b013e3181ab858a 19707128

[15] Drewry AM Samra N Skrupky LP Fuller BM Compton SM Hotchkiss RS . Persistent lymphopenia after diagnosis of sepsis predicts mortality. Shock. (2014) 42:383–91. doi: 10.1097/shk.0000000000000234 25051284 PMC4362626

[16] Scicluna BP van Vught LA Zwinderman AH Wiewel MA Davenport EE Burnham KL . Classification of patients with sepsis according to blood genomic endotype: a prospective cohort study. Lancet Respir Med. (2017) 5:816–26. doi: 10.1016/s2213-2600(17)30294-1 28864056

[17] Hotchkiss RS Moldawer LL . Parallels between cancer and infectious disease. N Engl J Med. (2014) 371:380–3. doi: 10.1056/NEJMcibr1404664 25054723

[18] Meisel C Schefold JC Pschowski R Baumann T Hetzger K Gregor J . Granulocyte-macrophage colony-stimulating factor to reverse sepsis-associated immunosuppression: a double-blind, randomized, placebo-controlled multicenter trial. Am J Respir Crit Care Med. (2009) 180:640–8. doi: 10.1164/rccm.200903-0363OC 19590022

[19] Kaukonen KM Bailey M Suzuki S Pilcher D Bellomo R . Mortality related to severe sepsis and septic shock among critically ill patients in Australia and New Zealand, 2000-2012. Jama. (2014) 311:1308–16. doi: 10.1001/jama.2014.2637 24638143

[20] Monneret G Venet F Kullberg BJ Netea MG . ICU-acquired immunosuppression and the risk for secondary fungal infections. Med Mycol. (2011) 49 Suppl 1:S17–23. doi: 10.3109/13693786.2010.509744 20718607

[21] Wiersinga WJ Leopold SJ Cranendonk DR van der Poll T . Host innate immune responses to sepsis. Virulence. (2014) 5:36–44. doi: 10.4161/viru.25436 23774844 PMC3916381

[22] Denzel C Riese J Hohenberger W Born G Köckerling F Tschaikowsky K . Monitoring of immunotherapy by measuring monocyte HLA-DR expression and stimulated TNFalpha production during sepsis after liver transplantation. Intensive Care Med. (1998) 24:1343–4. doi: 10.1007/s001340050775 9885893

[23] Monneret G Venet F . Sepsis-induced immune alterations monitoring by flow cytometry as a promising tool for individualized therapy. Cytometry B Clin Cytom. (2016) 90:376–86. doi: 10.1002/cyto.b.21270 26130241

[24] Döcke WD Höflich C Davis KA Röttgers K Meisel C Kiefer P . Monitoring temporary immunodepression by flow cytometric measurement of monocytic HLA-DR expression: a multicenter standardized study. Clin Chem. (2005) 51:2341–7. doi: 10.1373/clinchem.2005.052639 16214828

[25] Monneret G Lafon T Gossez M Evrard B Bodinier M Rimmelé T . Monocyte HLA-DR expression in septic shock patients: insights from a 20-year real-world cohort of 1023 cases. Intensive Care Med. (2025) 51:1820–32. doi: 10.1007/s00134-025-08110-w 40986015 PMC12504386

[26] Demaret J Walencik A Jacob MC Timsit JF Venet F Lepape A . Inter-laboratory assessment of flow cytometric monocyte HLA-DR expression in clinical samples. Cytometry B Clin Cytom. (2013) 84:59–62. doi: 10.1002/cyto.b.21043 22987669

[27] Bermejo-Martin JF Cavaillon JM Bouma H . Thirty years of mHLA-DR research in septic shock: lessons and caveats for clinical translation. Intensive Care Med. (2026) 52:138–41. doi: 10.1007/s00134-025-08220-5 41273370

[28] Giamarellos-Bourboulis EJ Kotsaki A Kotsamidi I Efthymiou A Koutsoukou V Ehler J . Precision immunotherapy to improve sepsis outcomes: the ImmunoSep randomized clinical trial. Jama. (2026) 335:775–86. doi: 10.1001/jama.2025.24175 41359996 PMC12687202

[29] Kotsaki A Pickkers P Bauer M Calandra T Lupse M Wiersinga WJ . ImmunoSep (Personalised Immunotherapy in Sepsis) international double-blind, double-dummy, placebo-controlled randomised clinical trial: study protocol. BMJ Open. (2022) 12:e067251. doi: 10.1136/bmjopen-2022-067251 36600424 PMC9772655

[30] Wu JF Ma J Chen J Ou-Yang B Chen MY Li LF . Changes of monocyte human leukocyte antigen-DR expression as a reliable predictor of mortality in severe sepsis. Crit Care. (2011) 15:R220. doi: 10.1186/cc10457 21933399 PMC3334765

[31] Cavaillon JM Adib-Conquy M . Bench-to-bedside review: endotoxin tolerance as a model of leukocyte reprogramming in sepsis. Crit Care. (2006) 10:233. doi: 10.1186/cc5055 17044947 PMC1751079

[32] López-Collazo E del Fresno C . Pathophysiology of endotoxin tolerance: mechanisms and clinical consequences. Crit Care. (2013) 17:242. doi: 10.1186/cc13110 24229432 PMC4059412

[33] Poehlmann H Schefold JC Zuckermann-Becker H Volk HD Meisel C . Phenotype changes and impaired function of dendritic cell subsets in patients with sepsis: a prospective observational analysis. Crit Care. (2009) 13:R119. doi: 10.1186/cc7969 19604380 PMC2750167

[34] Waeckel L Venet F Gossez M Monard C Rimmelé T Monneret G . Delayed persistence of elevated monocytic MDSC associates with deleterious outcomes in septic shock: a retrospective cohort study. Crit Care. (2020) 24:132. doi: 10.1186/s13054-020-02857-y 32264937 PMC7137224

[35] Le Tulzo Y Pangault C Gacouin A Guilloux V Tribut O Amiot L . Early circulating lymphocyte apoptosis in human septic shock is associated with poor outcome. Shock. (2002) 18:487–94. doi: 10.1097/00024382-200212000-00001 12462554

[36] Hotchkiss RS Tinsley KW Swanson PE Schmieg Jr RE Hui JJ Chang KC . Sepsis-induced apoptosis causes progressive profound depletion of B and CD4+ T lymphocytes in humans. J Immunol. (2001) 166:6952–62. doi: 10.4049/jimmunol.166.11.6952 11359857

[37] Xiao Y Xu L Jiang Y Wang Q Deng J Luo Z . Immunomodulatory therapy for sepsis: from pathophysiological mechanisms to precision treatment. Front Cell Infect Microbiol. (2025) 15:1746254. doi: 10.3389/fcimb.2025.1746254 41756408 PMC12932498

[38] Saavedra-Torres JS Pinzón-Fernández MV Nati-Castillo HA Cadena Correa V Lopez Molina LC Gaitán JE . Immunodynamic disruption in sepsis: mechanisms and strategies for personalized immunomodulation. Biomedicines. (2025) 13:2139. doi: 10.3390/biomedicines13092139 41007702 PMC12467355

[39] Venet F Rimmelé T Monneret G . Management of sepsis-induced immunosuppression. Crit Care Clin. (2018) 34:97–106. doi: 10.1016/j.ccc.2017.08.007 29149944

[40] Hotchkiss RS Colston E Yende S Crouser ED Martin GS Albertson T . Immune checkpoint inhibition in sepsis: a Phase 1b randomized study to evaluate the safety, tolerability, pharmacokinetics, and pharmacodynamics of nivolumab. Intensive Care Med. (2019) 45:1360–71. doi: 10.1007/s00134-019-05704-z 31576433 PMC9006384

[41] Patil NK Guo Y Luan L Sherwood ER . Targeting immune cell checkpoints during sepsis. Int J Mol Sci. (2017) 18:2413. doi: 10.3390/ijms18112413 29135922 PMC5713381

[42] Sari MI Ilyas S . The expression levels and concentrations of PD-1 and PD-L1 proteins in septic patients: a systematic review. Diagnostics (Basel). (2022) 12:2004. doi: 10.3390/diagnostics12082004 36010357 PMC9407082

[43] Dong X Liu Q Zheng Q Liu X Wang Y Xie Z . Alterations of B cells in immunosuppressive phase of septic shock patients. Crit Care Med. (2020) 48:815–21. doi: 10.1097/ccm.0000000000004309 32304414

[44] Zhang Y Wu F Li Y Liu J Zhu L Zhang M . SREBF1 mediates immunoparalysis of dendritic cells in sepsis by regulating lipid metabolism and endoplasmic reticulum stress. Cell Commun Signal. (2025) 23:286. doi: 10.1186/s12964-025-02295-9 40524162 PMC12172212

[45] Reizine F Grégoire M Lesouhaitier M Coirier V Gauthier J Delaloy C . Beneficial effects of citrulline enteral administration on sepsis-induced T cell mitochondrial dysfunction. Proc Natl Acad Sci USA. (2022) 119:e2115139119. doi: 10.1073/pnas.2115139119 35173051 PMC8872724

[46] Domínguez-Andrés J Novakovic B Li Y Scicluna BP Gresnigt MS Arts RJW . The itaconate pathway is a central regulatory node linking innate immune tolerance and trained immunity. Cell Metab. (2019) 29:211–220.e5. doi: 10.1016/j.cmet.2018.09.003 30293776

[47] Huang J Xia Y Zhang Y Zhang C Chen Z Hou X . Extracellular vesicle-associated PD-L1 induces CD4(+) T cell dysfunction and contributes to immunosuppression in sepsis. Cell Commun Signal. (2026) 24:174. doi: 10.1186/s12964-026-02724-3 41668092 PMC12990564

[48] Xiang J Wang Y Shi L Qiu J Gan L Xu Z . Optimal timing of PD-1/PD-L1 blockade protects organ function during sepsis. Inflammation. (2025) 48:975–86. doi: 10.1007/s10753-024-02113-3 PMC12234622 39174864

[49] Chen Y Guo DZ Zhu CL Ren SC Sun CY Wang Y . The implication of targeting PD-1:PD-L1 pathway in treating sepsis through immunostimulatory and anti-inflammatory pathways. Front Immunol. (2023) 14:1323797. doi: 10.3389/fimmu.2023.1323797 38193090 PMC10773890

[50] Giamarellos-Bourboulis EJ Bauer M Sweeney TE . Endotype trajectories: better understanding for better sepsis management. Intensive Care Med. (2026) 52:542–4. doi: 10.1007/s00134-026-08323-7 41661289

[51] Davenport EE Burnham KL Radhakrishnan J Humburg P Hutton P Mills TC . Genomic landscape of the individual host response and outcomes in sepsis: a prospective cohort study. Lancet Respir Med. (2016) 4:259–71. doi: 10.1016/s2213-2600(16)00046-1 26917434 PMC4820667

[52] Sweeney TE Azad TD Donato M Haynes WA Perumal TM Henao R . Unsupervised analysis of transcriptomics in bacterial sepsis across multiple datasets reveals three robust clusters. Crit Care Med. (2018) 46:915–25. doi: 10.1097/ccm.0000000000003084 29537985 PMC5953807

[53] Rademaker E van Amstel RBE El Bouhaddani S Bonten MJM Derde LPG van Vught L . Temporal robustness of biomarker-based classification algorithms for sepsis. Intensive Care Med. (2026) 52:22–30. doi: 10.1007/s00134-025-08218-z 41324692 PMC12852201

[54] Komorowski M Celi LA Badawi O Gordon AC Faisal AA . The Artificial Intelligence Clinician learns optimal treatment strategies for sepsis in intensive care. Nat Med. (2018) 24:1716–20. doi: 10.1038/s41591-018-0213-5 30349085

[55] Samuelsen A Lehman E Burrows P Bonavia AS . Time-dependent variation in immunoparalysis biomarkers among patients with sepsis and critical illness. Front Immunol. (2024) 15:1498974. doi: 10.3389/fimmu.2024.1498974 39712015 PMC11659229

[56] Monneret G Payen D . Immunostimulation in critically ill patients: do not forget to consider the timing, stratification, and monitoring. Intensive Care. (2024) 14:75. doi: 10.1186/s13613-024-01302-5 38758465 PMC11101384

[57] Zhang R Long F Wu J Tan R . Distinct immunological signatures define three sepsis recovery trajectories: a multi-cohort machine learning study. Front Med (Lausanne). (2025) 12:1575237. doi: 10.3389/fmed.2025.1575237 40313554 PMC12045099

[58] Wheelwright J Halstead ES Knehans A Bonavia AS . Ex vivo endotoxin stimulation of blood for predicting survival in patients with sepsis: a systematic review. CHEST Crit Care. (2023) 1. doi: 10.1016/j.chstcc.2023.100029 38148988 PMC10751038

[59] Cohen J . The immunopathogenesis of sepsis. Nature. (2002) 420:885–91. doi: 10.1038/nature01326 12490963

[60] Andaluz-Ojeda D Bobillo F Iglesias V Almansa R Rico L Gandía F . A combined score of pro- and anti-inflammatory interleukins improves mortality prediction in severe sepsis. Cytokine. (2012) 57:332–6. doi: 10.1016/j.cyto.2011.12.002 22197776

[61] Ruiz-Rodríguez JC Chiscano-Camón L Ruiz-Sanmartin A Costa-Allué N Bajaña I Nicolas-Morales P . Critical hypercytokinemia in sepsis and septic shock: identifying interleukin-6 thresholds beyond which mortality risk exceeded survival probability. J Clin Med. (2026) 15. doi: 10.3390/jcm15031057 41682739 PMC12897864

[62] Fajgenbaum DC June CH . Cytokine storm. N Engl J Med. (2020) 383:2255–73. doi: 10.1056/NEJMra2026131 33264547 PMC7727315

[63] Mera S Tatulescu D Cismaru C Bondor C Slavcovici A Zanc V . Multiplex cytokine profiling in patients with sepsis. Apmis. (2011) 119:155–63. doi: 10.1111/j.1600-0463.2010.02705.x 21208283

[64] Bozza FA Salluh JI Japiassu AM Soares M Assis EF Gomes RN . Cytokine profiles as markers of disease severity in sepsis: a multiplex analysis. Crit Care. (2007) 11:R49. doi: 10.1186/cc5783 17448250 PMC2206478

[65] Fan JB Li QY Feng XF Huang SY Wang R Liao FY . The "cytokine storm" in infection and sepsis: win the battle but lose the war. Mil Med Res. (2026) 12:95. doi: 10.1186/s40779-025-00678-0 41521316 PMC12794442

[66] Conway Morris A Datta D Shankar-Hari M Stephen J Weir CJ Rennie J . Cell-surface signatures of immune dysfunction risk-stratify critically ill patients: INFECT study. Intensive Care Med. (2018) 44:627–35. doi: 10.1007/s00134-018-5247-0 29915941 PMC6006236

[67] Liu X Jiang X Zhang X Hu Q Yu X Sun J . Methyl-CpG-binding domain protein 2 epigenetically represses monocyte HLA-DR expression and promotes immune paralysis in HBV-related acute-on-chronic liver failure. Front Immunol. (2026) 17:1694659. doi: 10.3389/fimmu.2026.1694659 41789066 PMC12956542

[68] Delsesto D Opal SM . Future perspectives on regulating pro-and anti-inflammatory responses in sepsis. Contrib Microbiol. (2011) 17:137–56. doi: 10.1159/000324030 21659751

[69] Viktorsson SA Turnbull IR . Sepsis in surgical patients: personalized medicine in the future treatment of sepsis. Surgery. (2024) 176:544–6. doi: 10.1016/j.surg.2024.03.042 38760228 PMC11246797

[70] Miller RR Lopansri BK Burke JP Levy M Opal S Rothman RE . Validation of a host response assay, SeptiCyte LAB, for discriminating sepsis from systemic inflammatory response syndrome in the ICU. Am J Respir Crit Care Med. (2018) 198:903–13. doi: 10.1164/rccm.201712-2472OC 29624409 PMC6835074

[71] Gouel-Chéron A Allaouchiche B Guignant C Davin F Floccard B Monneret G . Early interleukin-6 and slope of monocyte human leukocyte antigen-DR: a powerful association to predict the development of sepsis after major trauma. PloS One. (2012) 7:e33095. doi: 10.1371/journal.pone.0033095 22431998 PMC3303782

[72] Inoue S Suzuki-Utsunomiya K Okada Y Taira T Iida Y Miura N . Reduction of immunocompetent T cells followed by prolonged lymphopenia in severe sepsis in the elderly. Crit Care Med. (2013) 41:810–9. doi: 10.1097/CCM.0b013e318274645f 23328259

[73] Pierrakos C Vincent JL . Sepsis biomarkers: a review. Crit Care. (2010) 14:R15. doi: 10.1186/cc8872 20144219 PMC2875530

[74] Reinhart K Meisner M Brunkhorst FM . Markers for sepsis diagnosis: what is useful? Crit Care Clin. (2006) 22:503–19. doi: 10.1016/j.ccc.2006.03.003 16893736

[75] Mira JC Gentile LF Mathias BJ Efron PA Brakenridge SC Mohr AM . Sepsis pathophysiology, chronic critical illness, and persistent inflammation-immunosuppression and catabolism syndrome. Crit Care Med. (2017) 45:253–62. doi: 10.1097/ccm.0000000000002074 27632674 PMC5243156

[76] Angus DC van der Poll T . Severe sepsis and septic shock. N Engl J Med. (2013) 369:840–51. doi: 10.1056/NEJMra1208623 23984731

[77] Zimmerman JJ Sullivan E Yager TD Cheng C Permut L Cermelli S . Diagnostic accuracy of a host gene expression signature that discriminates clinical severe sepsis syndrome and infection-negative systemic inflammation among critically ill children. Crit Care Med. (2017) 45:e418–25. doi: 10.1097/ccm.0000000000002100 27655322

[78] Marshall JC Cook DJ Christou NV Bernard GR Sprung CL Sibbald WJ . Multiple organ dysfunction score: a reliable descriptor of a complex clinical outcome. Crit Care Med. (1995) 23:1638–52. doi: 10.1097/00003246-199510000-00007 7587228

[79] Boomer JS Green JM Hotchkiss RS . The changing immune system in sepsis: is individualized immuno-modulatory therapy the answer? Virulence. (2014) 5:45–56. doi: 10.4161/viru.26516 24067565 PMC3916383

[80] Badero OJ Omokore O Oore-Ofe O Osibowale B Quadri I Olayinka T . Immune endotype-guided precision immunotherapy in sepsis: from transcriptomics to bedside application. Cureus. (2026) 18:e106312. doi: 10.7759/cureus.106312 41938673 PMC13044672

[81] Nalos M Santner-Nanan B Parnell G Tang B McLean AS Nanan R . Immune effects of interferon gamma in persistent staphylococcal sepsis. Am J Respir Crit Care Med. (2012) 185:110–2. doi: 10.1038/s41591-018-0213-5 22210794

[82] Pinder EM Rostron AJ Hellyer TP Ruchaud-Sparagano MH Scott J Macfarlane JG . Randomised controlled trial of GM-CSF in critically ill patients with impaired neutrophil phagocytosis. Thorax. (2018) 73:918–25. doi: 10.1136/thoraxjnl-2017-211323 30064991 PMC6166597

[83] Francois B Jeannet R Daix T Walton AH Shotwell MS Unsinger J . Interleukin-7 restores lymphocytes in septic shock: the IRIS-7 randomized clinical trial. JCI Insight. (2018) 3. doi: 10.1172/jci.insight.98960 29515037 PMC5922293

[84] Yang Y Zhang Y Wu J Liu Y Lei X . Decoding immune low-response states in sepsis: single-cell and 3D spatial transcriptomic insights into immunoparalysis. Front Immunol. (2025) 16:1696914. doi: 10.3389/fimmu.2025.1696914 41268561 PMC12626914

[85] Seymour CW Kennedy JN Wang S Chang CH Elliott CF Xu Z . Derivation, validation, and potential treatment implications of novel clinical phenotypes for sepsis. Jama. (2019) 321:2003–17. doi: 10.1001/jama.2019.5791 31104070 PMC6537818

[86] Maddali MV Churpek M Pham T Rezoagli E Zhuo H Zhao W . Validation and utility of ARDS subphenotypes identified by machine-learning models using clinical data: an observational, multicohort, retrospective analysis. Lancet Respir Med. (2022) 10:367–77. doi: 10.1016/s2213-2600(21)00461-6 35026177 PMC8976729

